# Development and Implementation of an Alternate Care Site During the COVID-19 Pandemic

**DOI:** 10.7759/cureus.10799

**Published:** 2020-10-05

**Authors:** Kurien Mathews, Mikhail Podlog, Josh Greenstein, Eric Cioè‐Peña, Bartholomew Cambria, Brahim Ardolic, Barry Hahn, Joseph Basile

**Affiliations:** 1 Emergency Medicine, Staten Island University Hospital, Staten Island, USA

**Keywords:** surge capacity, resource allocation, administration

## Abstract

Introduction

With the rampant spread of severe acute respiratory syndrome coronavirus 2 (SARS-CoV-2) and the subsequent pandemic of coronavirus disease 2019 (COVID-19), the need for medical resources has never been greater. In recent history, the deployment of surge medical facilities and their importance in improving the provision of crisis care became relevant. The primary objective of this study was to describe the development and implementation of an alternate care site (ACS) during the COVID-19 pandemic.

Methods

This was a retrospective, single-center study that was conducted between April 7, 2020, and May 26, 2020, of adult patients from a primary facility admitted to an ACS, labeled Staten Island University Hospital East (SIUH-E). These select patients met specific inclusion criteria for SIUH-E before transfer.

Results

During the operational course of SIUH-E, 813 patients were screened and 203 patients were accepted for transfer. Of the patients admitted to SIUH-E, 120 (59%) were male. The mean age was 63 years (SD = 13.91). The mean length of stay was 3.93 days (SD = 3.94). Among discharged patients, 179 (88%) were discharged to home or another long-term facility, whereas 24 (12%) patients required a transfer back to the main campus.

Conclusions

In this study, we describe the development and implementation of an alternate care surge facility during the COVID-19 pandemic. SIUH-E played a vital role in effectively caring for select COVID-19 patients, which allowed the primary facilities to treat a greater volume of higher acuity patients. The combined efforts of the state and hospital were able to create and sustain a safe, practical alternative care facility.

## Introduction

Background

With the rampant spread of severe acute respiratory syndrome coronavirus 2 (SARS-CoV-2) and the subsequent pandemic of coronavirus disease 2019 (COVID-19), the need for medical resources has never been greater. As of June 2020, the total cases in the New York City (NYC) Region are more than 210,000, whereas deaths confirmed and related to COVID-19 tower at 6,488. During the peak of the pandemic, cases within the NYC region spiked up to 1,700 patients hospitalized in a single day, with 900 patient deaths per day. This volume of patients placed a tremendous burden on the city's healthcare infrastructure. Staten Island University Hospital (SIUH), which is part of the Northwell Health System, was at the epicenter and experienced a significant patient burden. Specifically, during March and April of 2020, where the case density was the highest, SIUH's intensive care unit operated at 300% capacity [1].

Relevance

In recent history, specifically with the disaster of Hurricane Katrina, the deployment of surge medical facilities and their importance in improving the provision of crisis care became relevant [2,3]. The COVID-19 pandemic poses a similar threat of overwhelming healthcare resources. As the number of cases increased in March and April 2020, the strain on the community and healthcare infrastructure became increasingly relevant. As a result, an alternate care site (ACS) was deployed for COVID-19 patients. This surge facility was established in Staten Island, New York, in partnership with the New York State (NYS) Governor’s Office to offload the volume of patients from SIUH North (SIUH-N) and South (SIUH-S) inpatient campuses. The goal of this site was to treat a select cohort of COVID-19 patients that required intermediate and longer-term inpatient therapy while allowing higher acuity and resource-intense patients to remain in the primary facilities.

Goals of the investigation

The primary objective of this study was to describe the development and implementation of an ACS during the COVID-19 pandemic.

## Materials and methods

Setting

This was a retrospective, single-center study that was conducted between April 7, 2020, and May 26, 2020, of patients admitted to an ACS, SIUH-East (SIUH-E). SIUH comprises two geographically distinct clinical sites. SIUH-N is a 700-bed academic, tertiary care center, with 97,000 Emergency Department (ED) patient visits per year. SIUH-S is a 200-bed urban community hospital, with 32,000 ED patient visits per year. These hospitals are located eight miles apart in Staten Island, New York, but share a single administration with unified oversight of operations, clinical quality, and doctor staffing. The local institutional review board approved the study.

Population

The study included patients ≥ 21 years of age who were admitted to either SIUH-N or SIUH-S with a diagnosis of COVID-19 based on a positive polymerase chain reaction (PCR) test. These select patients met specific inclusion criteria for SIUH-E before transfer (Table 1).

**Table 1:** Staten Island University Hospital East Inclusion Criteria for Transfer NC, nasal cannula

Inclusion Criteria for Transfer
Weight less than 350 lbs
Not bed-bound
Oxygen saturation greater than 90% on 4L NC
Inpatient stay of greater than 48 hours
Cannot have O_2_ requirements greater than 4L on NC
No hemodialysis or peritoneal dialysis
No transfusion, chemotherapy, immunoglobulin, or radiation therapy requirements
No behavioral issues
Does not require continuous telemetry monitoring

Protocol

Data were obtained on patient encounters through a query of Tableau (Salesforce, Seattle, Washington) and Excel (Microsoft Corporation, Redmond, Washington) generated with non-patient identifiers. Data were maintained on Research Electronic Data Capture (REDCap), a secure, web-based application designed to support data capture for research studies.

Measurements

Demographic variables extracted included age, gender, race (African American, Asian, Native American, Multiracial, White, and unknown), and ethnicity (Hispanic, not Hispanic, and unknown). Comorbidities included hypertension (HTN), coronary artery disease, congestive heart failure, asthma, chronic obstructive pulmonary disease, obstructive sleep apnea, chronic renal failure, end-stage renal disease, cirrhosis, hepatitis B, hepatitis C, diabetes mellitus (DM), and smoking history. In addition, data regarding hospital length of stay (LOS), ACS LOS, discharge to home, discharge to a skilled nursing facility, and transfer back to SIUH-N from ACS were also obtained.

Data analysis 

The data were analyzed using descriptive statistical methods and expressed as frequency counts and percentages for categorical variables.

## Results

During the operational course of SIUH-E, 813 patients were screened and 203 patients were accepted for transfer. Of the patients admitted to SIUH-E, 120 (59%) were male and 83 (41%) were female. The mean age of patients was 63 years (SD = 13.91). Among all patients, 75(37%) patients were ≥ 65 years of age. HTN (n = 109 [54%]) and DM (n = 69 [34%]) were the most frequent comorbidities for patients transferred to SIUH-E. Nearly 45 (22%) of patients had a current or previous history of smoking. A full description of demographic characteristics and comorbidities can be seen in Table 2.

**Table 2:** Baseline Characteristics of Patients Admitted to Staten Island University Hospital East

Baseline Characteristics of Patients (N = 203)	
Demographic Information	
Age (mean, SD)	63, 13
Sex	
Male	120 (59%)
Female	83 (41%)
Race	
White	105 (52%)
Other/multiracial	55 (27%)
African American/Black	24 (12%)
Asian	10 (5%)
Unavailable/Unknown	6 (3%)
Native American/Alaskan	3 (1%)
Ethnicity	
Not Hispanic or Latino	150 (74%)
Hispanic or Latino	40 (20%)
Unknown/declined	13 (6%)
Smoking history	
Current smoker	26 (13%)
Former smoker	19 (9%)
Never smoked	158 (78%)
Comorbidities	
Cardiovascular	
Hypertension	109 (53%)
Coronary artery disease	28 (14%)
Congestive heart failure	17 (8%)
Chronic respiratory disease	
Chronic obstructive pulmonary disease	21 (10%)
Asthma	11 (5%)
Obstructive sleep apnea	6 (3%)
Kidney disease	
Chronic renal failure	17 (8%)
End-stage renal disease	0
Liver disease	
Cirrhosis	0
Hepatitis B	1 (0.5%)
Hepatitis C	0
Metabolic disease	
Diabetes mellitus	69 (34%)

The mean initial hospital LOS before transfer to SIUH-E was 6.86 days (SD = 5.95). The mean SIUH-E LOS was 3.93 days (SD = 3.94). Among discharged patients, 179 (82%) were discharged to home or another long-term facility, whereas 24 (12%) patients required a transfer back to the main SIUH campus. Of the patients requiring transfer back, the majority of patients (14 [58%]) needed transfer for hypoxia. No patients required intubation prior to transfer. A summary of the LOS and final disposition is displayed in Table 3.

**Table 3:** Length of Stay and Disposition of Patients Admitted to Staten Island University Hospital-East

Length of Stay and Disposition of Patients		
Length of stay prior to transfer (days)		
Min	1	
Max	35	
Mean	7	
Median	5	
Length of stay at Staten Island University Hospital East (days)		
Min	1	
Max	22	
Mean	4	
Median	3	
Disposition		
Discharge	179	88%
Transfer to primary facility	24	12%

## Discussion

To respond to the surge of patients seen during the COVID-19 pandemic, the NYS government identified potential ACS. SIUH-E was designated as an ACS with the mandate to care for stable COVID-19 inpatients who were not suitable for discharge. This goal of this study was to describe the development and implementation of SIUH-E this ACS proved to be an excellent model to meet patient surges. The combined logistical support of the state and the clinical support of a healthcare institution was able to maintain a high level of patient care without sacrificing quality.

The concept of a field hospital generally invokes the idea of a remote location with minimal staffing and logistical support. Our model emphasized the need for a facility near a primary healthcare institution. This deployment demonstrated a seamless integration of inpatient computerized provider order entry and medical record, maintenance of patient's medication and patient regiment, laboratory, and radiology services. This also increased our inpatient units' capacity for higher acuity patients. Since the physical infrastructure of these units was already present, it only needed to be outfitted to resemble our inpatient processes. This obviated the need for tents, water, heat, sewage, power, and other infrastructure, which generally constitute a significant portion of deployment and maintenance effort.

Demographics

The patient demographics and comorbidities were similar to other patients with COVID-19 throughout NYC [1]. The overall population of Hispanics and African Americans on Staten Island is 17.9% and 11.8%, respectively, according to the United States Department of Commerce 2013 census [4]. The racial makeup of COVID patients treated at the ACS was similar to the ratio of Hispanic and African American individuals living in Staten Island. However, it should be noted a higher proportion of Hispanics and African Americans were affected by COVID-19 overall in NYC.1 Moreover, the mean age of patients admitted to the ACS was greater than 60 years, with a higher proportion of males.

Facility description

**Figure 1:**
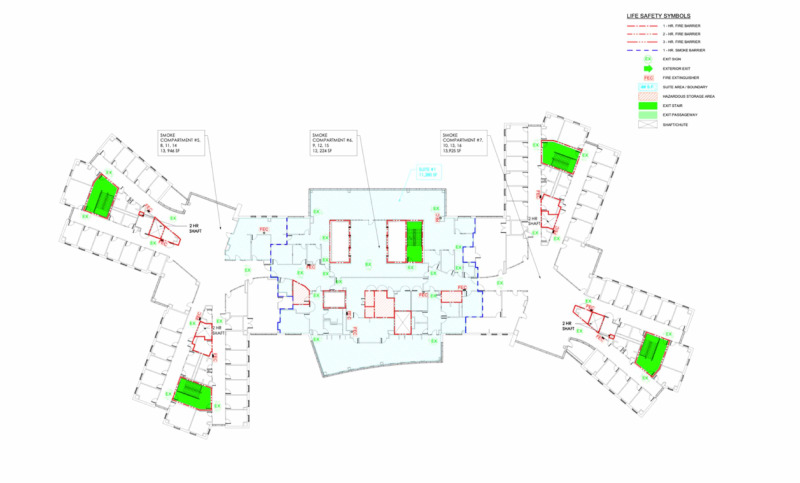
Schematic of a Standard Floor in the Alternate Care Site (SIUH-East) SIUH, Staten Island University Hospital East

The NYS Governor’s Office designated the newly constructed South Beach Psychiatric Facility as the location for SIUH-E. The facility’s location was approximately half a mile east of the SIUH-N campus. This facility was structured to be similar to a Hybrid Care ACS Level-2 Model, as outlined in the Centers for Disease Control and Prevention (CDC) guidelines [5]. The NYS Governor’s Office began necessary structural modifications to the site within seven days from the time the Northwell team was tasked to start the project. Figure 1 is a schematic of a standard floor in this facility. Each unit or wing contained 27 patient care rooms. Overall, the facility consisted of four levels with two units per floor.

Even with a newly built facility, several limitations existed due to the fact that the facility was initially created as a psychiatric center. First, there was a lack of oxygen supply in the building. The original accommodations were made smaller than traditional hospital rooms and with smaller entryways. Also, sinks and bathrooms were shared in a communal area for each wing of the unit. Finally, each room included just a single electrical outlet.

Managing the oxygen requirements of patients at the ACS was challenging. Oxygen concentrators, with a capacity of up to 5 liters per minute, were positioned in each patient care area, allowing for the use of supplemental oxygen. Additionally, oxygen tanks ranging from 15 to 250 liters were stored on-site for patients with additional oxygen requirements. This remedied the need for the installation of a centralized oxygen source. We were able to provide supplemental oxygen support for limited periods, especially during physical therapy, but there were substantial issues with maintaining a significant quantity of oxygen storage. Furthermore, delivering high flow oxygen would have required a negative pressure system in the ACS, and therefore high-flow O2 was not feasible in this space.

Because of the room dimensions, standard hospital beds could not be used, and, instead, smaller beds were utilized. These smaller beds were also not equipped with bed rails, resulting in limitations on accepting patients with a higher basal metabolic index and increasing the potential for falls. From an operational perspective, maintaining safety for patients that had limited mobility or were bed-bound became a significant challenge. All potential quality issues regarding patient falls were addressed as part of our quality review measures and improved by increased use of bed alarms and more robust fall protocols.

Facility setup

The COVID-19 facility was staged into cold, hot, and warm zones, with each zone separated by doors. The staged areas were instituted to comply with infection prevention and control recommendations from the CDC [6]. Surgical masks were mandatory within all zones of the SIUH-E, and N-95 masks were required within the hot zone. Both the NYS Governor’s Office and clinical command center operations were conducted within the cold zone. All patient care was performed in the hot zone. All medical supplies, telecommunications, oxygen supply, electronic medical records, and the pharmacy were also contained within the hot zone. All activities in the hot zone required full personal protective equipment (PPE). PPE included N-95 masks, gloves, gowns, hats, and goggles. The warm zone contained the on-call rooms along with the donning area before entering the hot zone. A separate doffing area was set up adjacent to the hot and warm zone for decontamination.

Command structure 

A dual command structure consisting of NYS and SIUH personnel was created during this period. NYS managed security, plant operations, environmental, and supplies. SIUH provided the clinical services, staffing, scheduling, and information technology (IT) support. It is our experience that this dual structure proved to be an effective means of operating a field hospital.

NYS command center

The Governor's office commissioned the NYS command center. It was led by Staten Island’s United States Congressman Max Rose, along with the 69th infantry division of the National Guard. Structural engineers were deployed to SIUH-E to address any deficiencies and shared joint projects with the NYC Javits Center surge facility. The NYS Department of Environmental Conservation communications division secured supplies from the state distribution supply chain.

SIUH command center 

SIUH's core team of physicians and nursing leadership managed clinical operations. The project was directed by the associate chair of the emergency department, a lead hospital administrator, a lead nurse administrator, and the executive director for SIUH. The team was tasked with the recruitment and deployment of clinical staff. The clinical operations command center was established adjacent to the inpatient COVID-19 unit at SIUH-E, from which the support staff and Northwell leadership operated. A liaison from the hospitalist service was present to facilitate transfers. This system assisted in identifying patients eligible for transfer.

Staffing

The staffing model resembled an inpatient unit team and consisted of a physician, advanced care practitioners (ACPs), nurses, and medical technicians. Physicians rounded on admitted patients daily. ACPs staffed the unit continuously. Due to the redeployment efforts by Northwell to re-allocate providers to hospitals with higher staffing needs due to patient volumes, we recruited physicians and ACPs from outpatient and inpatient specialties to staff SIUH-E. This provided a sufficient physician to ACP staffing ratio for our inpatient population. Staff scheduling was coordinated by the clinical director, nursing director, and non-clinical project managers. These managers were appointed specifically for SIUH-E. All staff was trained in PPE use to the appropriate level of patient care and facility access.

Within the hot zone, a clinical staff member was responsible for initiating and determining the scope of a rapid response. A rapid response was defined as a deterioration in vital signs with respiratory rate > 26 bpm, systolic blood pressure < 90 mm Hg, oxygen saturation < 90% on 5L, change in mental status, falls, or increasing intensity of medical resource use, as described previously in our results. The facility maintained a continuous rapid response coverage with an emergency medicine (EM) physician. Each EM physician was stationed in the warm zone for 24-hour intervals. They were available to provide critical care services for airway needs, circulatory support, and determination of patients requiring transfer to a higher level of care at SIUH-N. All EM physicians were equipped with video laryngoscopy and bedside point-of-care US capabilities along with a Northwell standardized code cart, airway box, and critical care medications needed for emergency airway management. Nurse staffing was provided through Northwell's human resources agency nurses. A single Emergency Medical Services crew with a paramedic was stationed at the facility to be available for transfers to and from the facility.

The National Guard was in constant communication with the command center. The guard's responsibility outside of their support and paramilitary role involved perimeter security, census verification, supply chain operations, and any tasks outside the realm of clinical support. Their coordinated efforts in the assembly of facility equipment, beds, and oxygen concentrators were essential in the rapid deployment of this project.

IT infrastructure

The deployment of an IT infrastructure was crucial to the development of SIUH-E and was accomplished by using Verizon as the local internet service provider. The entire facility was outfitted with internet capabilities within 72 hours of project initiation. The facility was equipped with routers and wireless internet access for all stationary and mobile workstations.

The ability to access inpatient records from across the Northwell system at SIUH-E provided a smooth transition for our clinicians' workflow. All computerized physician order entry, previous medical records, and picture archiving and communications system used across SIUH was available at SIUH-E. This integration enabled enterprise-related non-clinical tasks to appear seamless with Voice over Internet Protocol (VoIP) and telecommunications, email, and inpatient patient tracking software.

Pharmacy

The ACS was prepared with an on-site pharmacy, and all medications were stored in an automated medication dispensing system. All pharmaceuticals containing controlled substances were held in compliance with Drug Enforcement Administration regulations [7] and placed at controlled room temperature or refrigerated. All medications, including critical care mediations, were issued from the primary pharmacy at SIUH-N. All stock levels were monitored in real time, and medicine was restocked by pharmacy staff via a courier twice per day.

Dietary

The facility did not have food preparation capabilities, outside of basic food warmers. SIUH-N's food and nutrition services provided two cold and one hot meal a day for the patients.

Supply chain

To ensure sufficient medical and general facility supplies, a provision chain was developed. It leveraged the existing structure of NYS disaster planning resources and the central storage of SIUH-N. Project managers were in charge of the supply chain who ensured that the facility was adequately stocked. The same challenges for equipment procurement and a steady supply chain were also present during the COVID-19 crisis in many parts of the country. The coordinated effort of NYS and Northwell allowed all PPE and patient supplies care to be addressed.

Patient care 

All patient care closely emulated an inpatient medical-surgical unit. Care included supportive and pharmaceutical therapy for COVID-19, along with the continuation of home medication regimens and the management of chronic conditions. Treatment plans mirrored Northwell's protocols that were developed during the COVID-19 pandemic (Appendix 1). In terms of diagnostics and testing, in-house electrocardiogram (ECG) capabilities were present. For plain radiographs and computed tomography (CT), patients were transported back to SIUH-N. Stat laboratory specimens were available to be transferred to the primary institution via a courier delivery of samples for scheduled and unscheduled intervals. Daily physical therapy and occupational therapy were performed with appropriate patients. Social work matters were channeled through the parent institution who ensured safe and timely discharge. Specialty consultation occurred by phone, and patients requiring extensive consultation or treatments were transferred back to the primary facility. Most consultations were performed over the phone, and expanding future consultation capabilities using video and audio telehealth options could be considered. This includes using telehealth as an adjunct to conversations with families that are crucial to post-discharge care.

For patient comfort, the units provided communal areas to allow for patient interaction. All doors to patients' rooms were intentionally kept open, as it increased clinician awareness of patients' respiratory status and offered the ability to address any emergent needs quickly. iPads for patient entertainment were available upon request.

Disposal of materials

The trash and biohazard disposal processes were similar to SIUH-N. The facility was equipped with foot pedal disposable trash cans, soiled linen baskets, and sharps containers. Environmental services cleaned any non-medical trash and soiled linens. SIUH contracted a waste removal agency for final disposal. All soiled utility was transported back to SIUH-N, and clean blankets, pillows, covers, and sheets were replenished daily. The primary facility’s medical waste management team transported sharps and biohazards to SIUH-N. These items were then disposed of according to routine procedures.

There are several limitations to this study. The development of this ACS was performed at a single institution in a specific region of the country and may not be generalizable. As with any retrospective review, there is an inherent limitation of such studies. Additionally, we did not review clinical data and hemodynamic parameters. Furthermore, we were not able to gather information on new thrombotic events, new or worsening renal function, or psychiatric developments as a COVID-19 sequela. This ACS was developed to resemble an inpatient patient care area. However, individual patients who required evaluations by surgery or intensive care were excluded from this group. Any rapidly deteriorating patients were assessed and treated by EM physicians. Finally, this ACS was established near a parent institution, which may not be feasible in other areas.

## Conclusions

In this study, we describe the development and implementation of an alternate care surge facility during the COVID-19 pandemic. SIUH-E played a vital role in effectively caring for select COVID-19 patients, which allowed SIUH-N and SIUH-S to treat a higher volume of higher acuity patients.

Our model emphasized the need for a facility near a primary healthcare institution. This deployment demonstrated a seamless integration of inpatient computerized provider order entry and medical record, maintenance of patient's medication and patient regimen, laboratory, and radiology services. The combined efforts of NYS and SIUH were able to create and sustain a safe, practical alternative care facility.

## Figures and Tables

**Figure 2:**
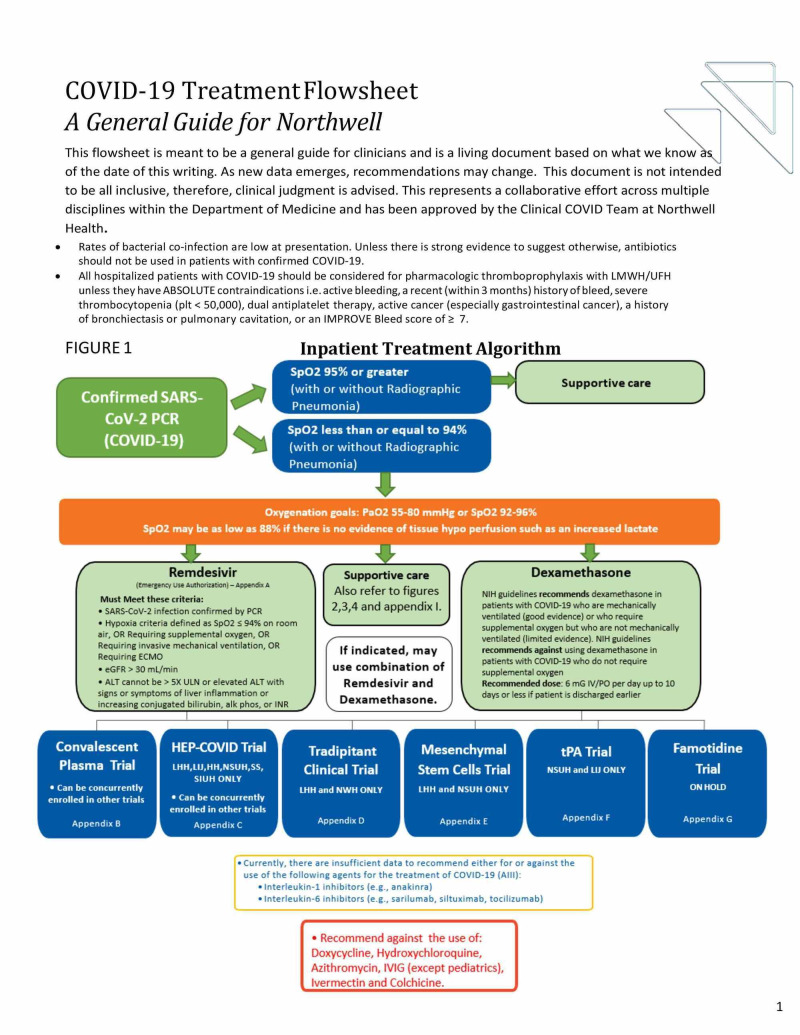
Northwell Health's Protocols for COVID-19 Pandemic Patients

**Figure 3:**
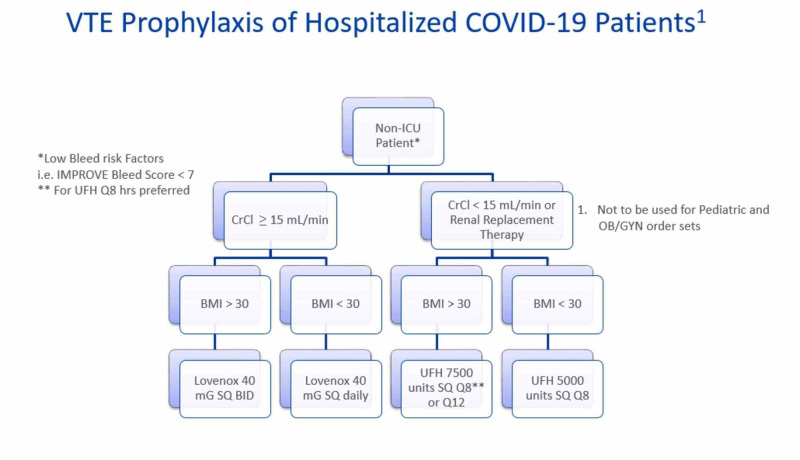
VTE prophylaxis of hospitalized COVID-19 patients VTE, venous thromboembolism

**Figure 4:**
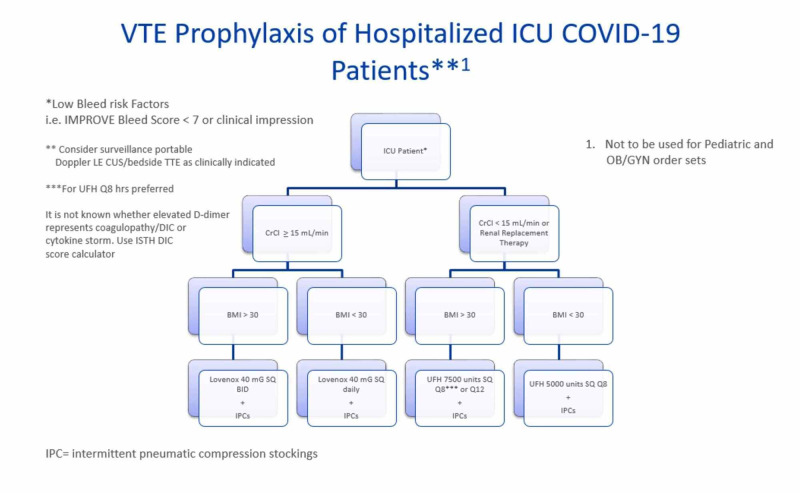
VTE prophylaxis of hospitalized ICU COVID-19 patients VTE, venous thromboembolism

**Figure 5:**
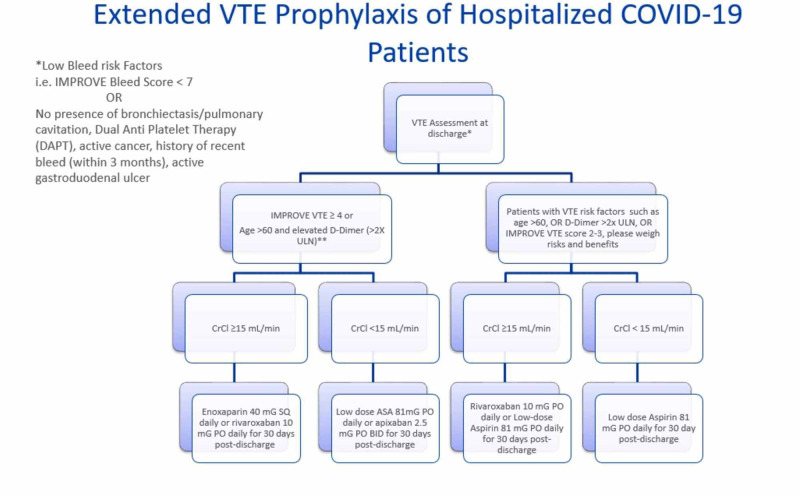
Extended VTE prophylaxis of hospitalized COVID-19 patients VTE, venous thromboembolism
